# Computer-Aided Sensor Development Focused on Security Issues

**DOI:** 10.3390/s16060759

**Published:** 2016-05-26

**Authors:** Andrzej Bialas

**Affiliations:** Institute of Innovative Technologies EMAG, Leopolda 31, Katowice 40-189, Poland; andrzej.bialas@ibemag.pl; Tel.: +48-606-747-864; Fax: +48-32-2007-701

**Keywords:** Common Criteria, security assurance, IT security development, intelligent sensor, design pattern, knowledge engineering, computer-aided security development

## Abstract

The paper examines intelligent sensor and sensor system development according to the Common Criteria methodology, which is the basic security assurance methodology for IT products and systems. The paper presents how the development process can be supported by software tools, design patterns and knowledge engineering. The automation of this process brings cost-, quality-, and time-related advantages, because the most difficult and most laborious activities are software-supported and the design reusability is growing. The paper includes a short introduction to the Common Criteria methodology and its sensor-related applications. In the experimental section the computer-supported and patterns-based IT security development process is presented using the example of an intelligent methane detection sensor. This process is supported by an ontology-based tool for security modeling and analyses. The verified and justified models are transferred straight to the security target specification representing security requirements for the IT product. The novelty of the paper is to provide a patterns-based and computer-aided methodology for the sensors development with a view to achieving their IT security assurance. The paper summarizes the validation experiment focused on this methodology adapted for the sensors system development, and presents directions of future research.

## 1. Introduction

The paper is focused on the security aspects of intelligent sensors. Generally, sensors are devices for measuring certain physical quantities and converting results into signals readable format for the observer or instrument. Additionally, intelligent sensors are able to process measured values. Intelligent sensors include sensor-, processing-, communicating facilities, and sometimes actuators. They can work autonomously or can be connected forming complex structures like sensor networks or systems. A sensor system can be a part of a more complex IT system. Apart from intelligent sensors, there are also smart sensors. To review the topic of how to distinguish intelligent sensors from smart ones [1], in this paper the author focuses on sensors which have IT product attributes, *i.e.*, the ability to process, store and transfer information. Such devices are equipped with microcontrollers. For these specific IT products information security is the key issue. 

Sensor and sensor system complexity and maturity have been growing and new sensor applications are emerging all the time. Intelligent sensors and sensor systems are extremely important to fulfil social and business objectives, including security- and safety-related objectives in many application domains, such as patient monitoring [2], the Internet of Things [3], smart cities [4], *etc.* Apart from public areas, like health services, services offered by the government, border control, communal applications, these solutions are crucial for high risk environments in the military sector, industry, aviation and other transport systems, traffic management, communications, navigation, identification, *etc.*

They are applied where the assessed risk is high or the appreciated information assets’ value is significant. For these applications high integrity and availability requirements are important.

Sensors and sensor systems should be secured like other information technology (IT) products or systems, but the sensor-specific limitations dealing with power sources, processing and communication capabilities should be taken into account. 

The responsible sensor applications require dependability (related to the system availability, reliability, and maintainability) and security assurance. Security assurance is the justified degree of confidence that the system meets its security requirements. It means that built-in security functions related to these requirements and representing security measures will counter effectively any threat when it occurs. The system architecture and its security features as well as the practices and procedures used should enforce the security policy rules related to these requirements.

The assurance standard [5] describes terminology and provides the basic assurance concepts, assurance techniques and framework. One of the most known assurance approaches is represented by the ISO/IEC 15408 Common Criteria (CC) [6,7,8,9]. The Common Criteria security assurance methodology provides rigorous rules for IT product development, independent product evaluation and rules for secure operation. This paper discusses the application of the Common Criteria assurance methodology to the development of intelligent sensors and is the continuation of the author’s earlier works.

Reference [10] reviews sensors and sensors networks security issues, including commonly known attacks. It presents the general sensor model and the corresponding security model, specifying assets, subjects, threats and security measures. The security model was validated on an intelligent mote-based medical sensor. In [11] the Common Criteria compliant security model for sensors is refined, introducing semiformal descriptions of the model items considered as the specification means patterns. These patterns can be used to build security models for different sensors. This methodology was validated on an intelligent sensor for early detection of methane. The further formalization of the Common Criteria compliant security model was performed by applying the knowledge engineering methodology [12]. The IT Security Development Ontology (ITSDO), encompassing security patterns for sensors, has been elaborated according to the basic knowledge engineering rules [13] and with the use of the Protégé Ontology Editor and Knowledge Acquisition System developed at Stanford University [14].

ITSDO encompasses security models only. These models are included in the security target (ST) or protection profile (PP) specifications (see a short CC primer in Section 1.1) according to the Common Criteria methodology. The ST specification is important, but it is only a part of the evidences that ought to be delivered for the evaluation. The finalized Common Criteria compliant, Modular, Open IT security Development Environment (CCMODE) R&D project [15] provides patterns for all evaluation evidences and computer support for the patterns-based development processes. CCMODE, co-financed by the EU within the European Fund of Regional Development, was performed by the author’s organization under the author’s leadership. The CCMODE project resulted in the following products:

- Patterns (including specification means, all evidences, documentation, procedures, *etc.*);
- Methodology and tools used to create and manage IT security development environments by different business organizations;
- Knowledge-related CC development and evaluation (a knowledgebase).

The results of the R&D on ITSDO [12] were used as the input to the CCMODE project, especially to elaborate knowledge engines for the IT security development process (see the short CC primer in Section 1.1), evaluation evidences patterns, security models and other supporting tools. 

The CCMODE IT security development environments can be applied for different IT products according to the Common Criteria methodology. The objective of the research presented in the paper is to adapt and validate this general-purpose methodology and the supporting tools with a view to using them in the intelligent sensors and sensors networks domain. 

The number of CC certified products in this domain is relatively low due to the existing barriers (complex character of the methodology, lack of knowledge, poor support for developers, high cost, *etc.*). The auxiliary objective is to help to overcome these barriers by disseminating domain knowledge and by providing a methodology and tools for the developers of secure products. 

The significance of the research presented in the paper lies in increasing the assurance of the developed sensors and sensors systems by applying the Common Criteria methodology and spreading it among the sensors technology developers. Please note that sensors and sensors systems are considered a specific class of IT products. Thanks to the CC methodology these products should be designed more rigorously, *i.e.*, better analyzed with respect to security, better tested, documented, managed, *etc.* Better controlled and more rigorous design will raise the sensors quality and security assurance, in the similar way as for any other IT products. The research is focused on two issues, a general one related to the CC methodology as a whole, and a specific one related to the use of this methodology in the intelligent sensors and sensors systems development.

### 1.1. Basic Common Criteria Methodology Terms Used in the Article

The Common Criteria methodology uses specific terms. An IT product is called a target of evaluation (TOE). It can be a hardware, software or IT system.

The second part of [6] contains components representing elementary security functional requirements (SFRs). They are used to express the behaviour of the TOE security functions, which represent countermeasures. The third part of [6] includes components expressing elementary security assurance requirements (SARs) for these security functions. Both sets of components, grouped by families, and families by classes, constitute a semiformal “language” to uniformly express security requirements for IT products.

Assurance is measured with the use of evaluation assurance levels (in the range EAL1 to EAL7). The assurance depends on the rigour applied to the security development process. The more rigorous is this process, the more precise are the applied engineering good practices (security analyses, testing, documentation), and the better is the organization of the development/production/maintenance environment—the more assurance has the IT product (the CC assurance paradigm). The given EAL represents the coherent package of security assurance requirements. The Common Criteria methodology [6] comprises three basic processes:

- The IT security development process, focused on the security analyses; the document called ST (security target) for the given TOE is worked out; the ST contains:
  ○ TOE overview and description sections;
  ○ Security problem definition (SPD), which specifies: the protected assets, subjects, threats, organizational security policies (OSPs), and security assumptions for the TOE;
  ○ Security objectives (SO) presenting how the security problem specified by the SPD is solved; the SO expresses generally the proposed security measures;
  ○ Security requirements (SFRs and SARs) present these solutions using the semiformal CC components;
  ○ TOE security functions for ST (TSF); they are the SFR implementation on the claimed EAL level;
- The TOE development process, focused on the elaboration of the CC-specific IT product documentation, embracing:
  ○ TOE architecture, its functional specification, design, security policy, implementation;
  ○ Live cycle definition, configuration management, product delivery, development process security, used tools and their options;
  ○ Tests specification, test depth and coverage;
  ○ Product manuals and procedures;
  ○ Vulnerability assessment of the TOE and its development site;
- The IT security evaluation process; it is performed by an independent, accredited security lab and finalized by certification; during this process the TOE and its evidences, *i.e.*, ST and the TOE documentation, are evaluated according to the CC methodology against the claimed EAL.

A protection profile (PP) can be considered a generic form of a security target. It provides an implementation independent specification of information assurance security requirements. Its content is similar to that of ST (SPD, SO, SFRs/SARs, no TSF), but more general. PPs are evaluated and registered. Security targets are developed on their basis. 

### 1.2. Current State of the Research Field

The review is focused on three basic issues related to the paper:

- Common Criteria application in sensors and sensors systems development;
- Patterns in security development;
- Computer support of the Common Criteria methodology.

During the review the author identified papers and reports related to the Common Criteria methodology used in the sensors and sensors networks development and a certain number of examples. The most relevant of them concern:

- General sensors network security aspects;
- Healthcare systems;
- Aircraft health monitoring systems;
- Safety-critical assets distribution systems;
- Transport, including motion sensors of digital tachographs;
- Products related to SCADA (Supervisory Control And Data Acquisition);
- Specialized firewalls used in control and automation systems, co-operating with sensors networks.

Reference [16] concerns the analysis of wireless sensor network (WSN) security, based on the regulations intended for wireless communication devices, like EN 50150 and MIL STD-188-220. It discusses WSN attacks, security measures countering these attacks, and provides a security evaluation and classification methodology for WSN protocols. The Common Criteria methodology is proposed as the validation methodology of security countermeasures in WSN systems. The countermeasures sufficiency (SFRs-based) and correctness (SARs-based) are evaluated. The countermeasures, identified as security objectives, are expressed with the use of the selected SFRs, e.g., “Source and destination identifier in each message” countermeasure is represented by the component of the CC families: FCO_NRO (Non-repudiation of origin), and “Membership control-bus guardian” by FIA_UID (User identification). 

A book chapter [17] is focused on the security and privacy issue in healthcare systems, including sensors and wireless sensors networks. The security and privacy threats are mapped to the commonly used countermeasures. The countermeasures are discussed with respect to the limitation of the sensors networks, for which it is difficult to apply advanced cryptographic techniques. It was remarked that assurance relies on engineering practices, development processes, operational issues, and can be evaluated according to the Common Criteria methodology. 

The first group of examples deals with e-Enabled aircraft [18]. The e-Enabled approach concerns the latest generation of airplanes and is applied to improve the safety and efficiency of air travel. Thanks to the advanced communication capabilities, including sensors systems based on a wireless network, e-Enabled aircraft can participate as intelligent nodes in the global information network. This network is the foundation of the following applications:

- Electronic Distribution of Software (EDS);
- Airplane Assets Distribution System (AADS);
- Air Health Monitoring and Management System (AHMMS);
- Air Traffic Control (ATC).

Common Criteria is used for the security analysis of an EDS system, identifying threats, deriving security objectives, requirements and security functions. This way the electronic distribution of software, cryptographic key and data between airplane and ground systems is secured. The system is based on the Public Key Infrastructure (PKI) services and digital signatures.

The paper [19] focuses on the use of wireless sensor networks (WSNs) for AHMMS. AHMMS is used:

- To monitor the health of airplane structures and board systems, which use embedded sensors;
- To give timely feedback to the flight control computer working on the board and to the airline ground server for health assessment.

The paper [20] concerns the Airplane Assets Distribution System (AADS). Assets encompass an authorized software (e.g., safety standards compliant software, firmware for intelligent sensors) and contents (data, security related data, unique identifiers, cryptographic keys, *etc.*) exchanged between airplanes and manufacturers, owners and servicers. Common Criteria is used for the security analysis of the communication channel between airplanes systems and between the airplane and the ground system. 

Similar communication channels are used in the automotive industry to load assets, *i.e.*, software or contents, to different embedded electronic control units in vehicles [21], tachographs, their motion sensors [22], *etc.*

The Common Criteria methodology can help to solve some difficult problems identified during research [21], like: building of the specialized high-assurance PKI, the use of formal methods for an end-to-end analysis of assets distribution systems, removing vulnerabilities and analyzing the impact of security on safety. 

The digital tachograph system [22,23] consists of a vehicle unit, motion sensor and a smart card used to log in to this unit. The Common Criteria standard is used to evaluate the security of tachograph systems [24,25,26]. 

The presentation [27] concerns protection against mileage frauds in cars and features a concept how to solve this problem. The Common Criteria methodology will be used to secure a communication channel from the ABS sensor to the car cockpit. The protection profile for an odometer is planned.

The paper [28] discusses a new generation of SCADA systems used to control virtual utilities, aggregating distributed resources, like microgrids, wind farms, fuel cells, *etc.* into single, centralized energy systems. Intelligent sensors can be used in control systems (field controllers) cooperating with SCADA. The author suggests that SCADA products should be evaluated with the use of the Common Criteria standard to avoid compromising the security or safety by these products. Security problems of SCADA are growing because SCADA products are often parts of critical information infrastructures.

The National Institute of Standards and Technology (NIST), in coordination with the Process Control Security Requirements Forum (PCSRF), initiated a research programme aimed at the risk reduction in industrial control systems based on the Common Criteria methodology [29]. The research results were IT security specifications for process control systems in the shape of a protection profile “System Protection Profile—Industrial Control Systems Version 1.0” [30].

The Common Criteria methodology is used in motion sensors [22]. For the above mentioned examples Common Criteria is used to secure systems co-operating with sensors systems. 

The Common Criteria methodology is rarely used in the sensor and sensor system certification process directly. Among many certified IT products (more than 2000) and registered protection profiles (more than 300) only few concern directly sensors and sensors systems, *i.e.*, motion sensors for digital tachographs and industrial control systems including sensors. There are also IT products indirectly related to sensors (the TOE does not comprise sensors), e.g., sensors systems supporting an IT product or working in its environment. Apart from these, the Common Criteria methodology is used for non-commercial purposes, but they are not well documented. 

The Common Criteria methodology is used as a development and evaluation methodology of sensors-related IT solutions, including very responsible products. Relevant examples were shown above. This situation is enforced by the current stage of the market development and is typical of the emerging domains of application of the Common Criteria methodology. The above mentioned papers present a need to use Common Criteria in the sensors systems development process. None of them presents the complete, CC-based development process. They do not mention evidence patterns or computer support either.

Design patterns can be understood as reusable, proven solutions to problems with respect to a specific context in the given domain of application [31]. The patterns include knowledge how to get this expected solution. The patterns are also used in the information technology, including its security. They may concern requirements, design and implementation [32]. They are specified in a formalized way, using different kinds of codes, models, ontologies, formalized descriptors, *etc.*

In [10,11] the author has defined the term “Common Criteria related security design patterns”, in compliance with the general patterns definition, but expressing the CC- specific issues, *i.e.*, different specification items, like CC-defined SFR/SAR components, as well as semiformal enhanced generics introduced by author. The CCMODE project [15] uses these patterns and introduces new ones, related to the evaluation evidences (documents) implied by SARs. Such a comprehensive set of patterns was a novel element, allowing for the automation of the evidences elaboration process. 

The paper [33] discusses the extension of the problem frame method to perform the CC-related security analyses. The supporting tool, based on UML/OCL (Unified Modeling Language/Object Constraint Language), is used (UML4PF) to perform modelling and analyses. The attacker model considering different attacker types was introduced. The method is focused on the security problem definition, and on this basis, the security objectives identification. 

Generally, to elaborate security targets, protection profiles and other evidences, developers are equipped with some guidelines, like: ISO/IEC 15446 [34]—for ST/PP or BSI guide [35]—for evidences up to EAL5. Some valuable practical hints about the evidence preparation, co-operation with external experts, work valuations and the certification itself are available in [9]. However, these guidelines have a general character and do not give any patterns to prepare the evidences.

There are a few software tools which support the development of evidences. The first and most known is Common Criteria (CC) Toolbox^TM^ [36], supporting the security target and protection profiles development.

GEST—a similar generator of security target templates is presented in [37]. It is based on the evaluated and certified security targets. 

Trusted Labs Security Editing Tool (TL SET) [38] helps the developer in writing and maintaining the security target and protection profiles according to CC. It also allows one to automatically generate documents in different formats.

Please note that these tools are focused on the ST and PP preparation only and do not support the elaboration of other evidence, e.g., dealing with the TOE design, life cycle, guidance, testing, vulnerability assessment. They are based on text processing and data bases and do not use advanced modelling or knowledge bases.

The sensors and sensors systems developers do not make sufficient use of the Common Criteria methodology. The sensors systems development is an emerging CC domain of application. In the emerging domains there are different barriers, like a lack of knowledge, tools and exemplars, low market demand, *etc.* Developers need some support. It can be provided by computer-aided specialized tools.

### 1.3. Research Motivation and Directions

The research presented in the paper is placed in the mainstream of research focused on the CC methodology implementation and improvement. This methodology is mature but is still being improved in the range of raising the design quality, facilitating the development and evaluation processes, decreasing the development cost and time. The general motivation of the author’s works is:

- To improve the IT security development process, thanks to the patterns-based approach;
- To minimize the barriers for developers, related to the lack of knowledge, methods and exemplars of evidences, *etc.*;

But the works presented in the paper are focused on the secure sensors and sensors systems development. The research presented in the paper uses three kinds of input:

- Theoretical foundation included in the author’s monograph [39], which includes the UML extension (stereotypes) to specify models of the IT security development framework in a formal way (syntax and semantics defined on mathematical principles), and the security models related to this framework (data and activity diagrams);
- The CCMODE project products [15]: methodology, tool and knowledge, to adapt them for the sensors systems domain of application;
- The research results from [10,11,12], to elaborate a library of predefined semiformal specification means for this domain.

The developed patterns-based, computer-aided methodology for sensor development is a novelty of this paper. The first kind of patterns encompasses evidences required by particular security assurance components on different EAL levels. The patterns are structured documents with predefined fields ready to be filled in with content related to the IT product and required by the Common Criteria standard. The developer is guided in elaborating evidence on the pattern basis. The second kind of patterns embraces predefined specification items—semiformal Common Criteria language—including not only components for the security requirements specification stage, but also enhanced generics defined by the author for other development stages. Thanks to the patterns, the evidence elaboration is easier and the evidence quality rises. Additional advantages are obtained by the automation of the evidence elaboration. The evidence content is introduced by the developer or is generated by the specialized document generator, which uses data from analytic/design aiding tools, knowledge base and the CC-related project management software. The software tool accelerates the evidence development and ensures the content reusability. 

The paper contributes to the sensors systems development by providing specialized, means and tools which comply with Common Criteria and rise quality and security assurance in this domain, particularly:

- Patterns and tools to implement this methodology, adapted to the sensors development domain;
- Knowledge concerning the Common Criteria assurance methodology and the sensors security.

The paper contains four sections: Section 1, above, discusses the Common Criteria basic issues, state of the art in the research domain, existing gaps and motivation for research. Section 2—the experimental section of the paper—presents the context and course of the validation experiment. Section 3 summarizes the achieved research results. Section 4 contains the conclusions of the paper.

## 2. Experimental Section

The experimental section provides a short introduction to the validation experiment, presenting shortly CCMODE Tools, implemented evaluation documents patterns and specification means patterns, the adaptation of the tool to sensor domain of application, and the validation plan. The validation experiment encompasses the tool setup according to the sensor project needs and the computer-aided ST development. The section concludes next steps to obtain a full design of the intelligent sensor. The validation experiment refers to the MEDIS sensor project described in [11].

### 2.1. General Features of CCMODE Tools

**Figure 1:**
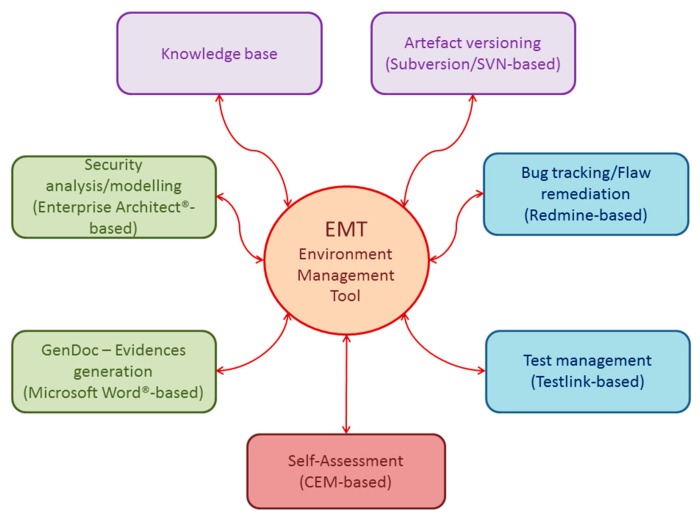
Block scheme of the CCMODE Tools suite.

CCMODE Tools is a specialized Computer-Aided Engineering (CAE) system focused on the Common Criteria-based IT product development. It supports even the most laborious and difficult operations in the IT product development process. Figure 1 shows the basic modules of CCMODE Tools [15].

“Environment Management Tool” (EMT) is the main module and the central entry point to CCMODE Tools. This is a specialized project manager focused on the Common Criteria related projects. It is responsible for the initialization/configuring of projects and their management in the life cycle. The project configuration complies with the chosen EAL package. The right evaluation evidence patterns (considering the additional or substituted SARs) are attached and the needed external tools are connected. Based on the Lightweight (Directory Access Protocol/Active Directory DAP/AD) EMT manages roles and users. For each project the life cycle, its phases, and processes are defined with the use of the predefined templates. EMT manages different project data, elaborated evidences, and different artefacts, e.g., implementation representation (code, electronic schemes), documentation, regulations, subcontractors, *etc.* This is also the entry point to other components shown in Figure 1. Using the D2RQ [40] technology, EMT integrates logically all CCMODE Tools components. 

The “Knowledge base” module manages the project knowledge and the CC-related knowledge. It includes the Common Criteria contents stored in the structured way, systems dictionaries, predefined patterns, security models, predefined life cycle models, and project-related data. It provides a context-sensitive help.

The “GenDoc—Evidences generation” (Microsoft Word^®^-based) module is designed to work out evidences on the basis of predefined patterns. The patterns are implemented as MS Word templates supported by software. GenDoc is the basic developer’s application. The developer, guided by the system, uses it directly and introduces basic contents to the given pattern to produce the corresponding evidence. A very helpful feature is that the contents can be loaded from the knowledge base and/or external systems (predefined values, analyses results). GenDoc allows to print the elaborated evidences or to generate empty patterns in the Microsoft Word^®^ format, which can be used outside GenDoc.

The “Security analysis/modelling” module is based on a plugin developed for Sparx Systems Enterprise Architect^®^ (EA). EA is a broadly used UML tool. The EA-plugin is used for security analyses and security modelling. The results are injected to the elaborated security targets or protection profiles. In addition, the EA-plugin provides input to evidences related to the decomposition, interfaces and tests of the IT product. 

The “Artefact versioning” module is based on the Subversion/SVN software [41]. It is responsible for versioning the project artefacts (including evidences). It supports configuration management according to the given EAL.

The “Bug tracking/Flaw remediation” module is based on the Redmine software [42]. It is used for bug tracking during the project progress and for flaws remediation according to Common Criteria (implementation of the ALC_FLR family requirements) after certification.

The “Test management” module is based on the Testlink software [43]. It is used for the tests development and management. It contains test plans and scenarios. It helps to specify functional tests and evidences related to the test depth and coverage.

The “Self-Assessment” module includes full implementation of the Common Criteria Evaluation Methodology (CEM). It can be used as a self-assessment, auxiliary tool by developers or it can be used separately by evaluators. It also contains auditing facilities which allow to assess conformance of the development environments with different standards, including CC.

CCMODE Tools supports traditional CC-related projects as well as site certification [44] projects.

### 2.2. Evaluation Evidences Patterns Implemented in CCMODE Tools

Developers should submit an IT product (TOE) and a set of evidences to an independent evaluation lab supervised by one of the Common Criteria certification bodies, which operates according to the Common Criteria Recognition Arrangement (CCRA) [7].

Evaluation evidences embrace:

- Documentation, e.g., configuration management plan;
- Documented results of independent investigations or observations conducted by the evaluators, e.g., a TOE vulnerability analysis report;
- Described behaviour or activities of people according to their roles in the TOE life cycle.

CCMODE Tools is equipped with a set of evaluation evidences patterns for all assurance components. Table 1 lists patterns related to the IT security development process. The most important is the Security Target pattern. It will be used as the basis for any sensor/sensors system project.

The TOE development process is based on two groups of patterns: those related to the environment (Table 2) where the IT product is developed and those related to the IT product itself (Table 3). Please note that Table 2 and Table 3 specify pattern families only, each SAR component has a dedicated pattern and the pattern names comply with assurance family names [6]. 

The items specified in Table 2 and Table 3 can be considered a short review of issues embraced by the Common Criteria standard. 

Some of these patterns will be exemplified during validation. 

### 2.3. Specification Means Patterns Implemented in CCMODE Tools

The above patterns specify structured documents whose elaboration is guided by the tool. Most of these patterns are filled in with a text with tables and graphics, however patterns shown in Table 1 need, additionally, semiformal specification means, *i.e.*, SFRs and SARs from CC and other descriptors defined by users and called “generics”. The author proposes [39] “enhanced generics”. They are defined as mnemonic names expressing common features, behaviours or actions related to IT security issues, like: subjects, objects, threats, assumptions, security policies, security objectives, and functions. They are “enhanced” since they are semiformal and have features comparable to CC components, allowing such operations as: parameterization, derivation, iteration, and refinement. 

An enhanced generic consists of four textual fields separated by dots, and the fourth field *Refinement* is optional:

*Family.Mnemonic.Description.Refinement*

The *Family* field expresses the generics taxonomy. The following groups are distinguished:

- Assets representing passive entities within the considered system, divided into subcategories:
  ― TOE related assets—marked DTO,
  ― Assets within the TOE operational environment (DEO),
- Subjects, representing active entities related to the TOE or its operational environment, including:
  ― Authorized subjects (SAU), e.g., user, administrator, process,
  ― Unauthorized entity (SNA), e.g., intruder,
  ― Non-human malicious entity (SNH), e.g., force majeure, failure,
- Threats, including:
  ― Direct attacks against the TOE (TDA),
  ― Attacks against the TOE operational environment (TEO),
- Assumptions, addressed to the TOE operational environment:
  ― Connectivity aspects (ACN),
  ― Personnel/organizational aspects (APR),
  ― Physical aspects (APH),
- Organizational security policies (OSPs) and security objectives have similar subcategories assigned:
  ― Control and information flow control (policy: PACC, objective: OACC),
  ― Identification and authentication (PIDA, OIDA),
  ― Accountability and security audit (PADT, OADT),
  ― Integrity (PINT, OINT),
  ― Availability (PAVB, OAVB),
  ― Privacy (PPRV, OPRV),
  ― Data exchange (PDEX, ODEX),
  ― Confidentiality (PCON, OCON),
  ― IT aspects of the TOE operational environment (PEIT, OEIT),
  ― Technical/physical aspects of the TOE operational environment (PEPH, OEPH),
  ― Security maintenance/management (PSMN, OSMN).

This version is marked “*Enhanced generics rev.3.1*”. When compared with the rev.3.0 [11], the new version has the generics related to the development site and security functions removed.

The *Mnemonic* field expresses very briefly, in a few letters, the generic meaning. 

The *Description* field contains one or a few sentences about the generic meaning, *i.e.*, the security features, behaviours or actions. *Description* can be supplemented by the optional *Refinement* field, e.g., 

*DTO.SensorID.*Unique identification number of an intelligent sensor.Identifier stored in a distinguished register written during the manufacturing process. 

Please note that the fields are separated by dots.

The *Description* field may have parameters in square brackets which represent any asset [*Dparam*] or any subject [*Sparam*]. The parameters may be left empty (meaning: “any possible”) or substituted (symbol: “<=”) by an appropriate asset or subject generic. Such parameterization enables iteratation of the enhanced generics. Here the given generics can be placed many times into the specifications with different parameters substituted which present different aspects of the same security issue. For example, a threat item with different-potential intruders attacking the same asset, or a threat with one intruder who attacks different assets, each one differently. Particular instances of the iterated enhanced generic are numbered with consecutive numbers placed in brackets. 

The paper [10] was focused on the identification of security issues for different kinds of sensors and sensors systems, according to the needs of the Common Criteria methodology. These issues encompass:

- Different kinds of protected assets:
  ― Basic assets, *i.e.*, sampled, processed, stored and transmitted data, and provided services,
  ― Assets whose availability allows the right operation of sensors (please note the restricted resources: energy, processing- and transmission capability),
  ― Security-related data, e.g., cryptographic keys, passwords, credentials, secrets,
  ― Assets placed within the TOE operational environment, encompassing all co-operating and mutually related IT entities, e.g., network gateways, monitoring central unit, common data base,
- Active entities (subjects):
  ― Legal users of a sensor or sensor system, user, admin, process,
  ― Legal actors participating in the life cycle processes performed within the site, service personnel,
  ― Intruders of different attack potential, force majeure, failure,
- Commonly known attacks against sensors and sensors systems—threats:
  ― Related to the data sampled/measured by the intelligent sensor, e.g., input data manipulation,
  ― Related to the data stored, processed and transferred by the intelligent sensor, e.g., illegal access, data manipulation,
  ― Exploiting vulnerabilities concerning sensors restricted resources, like power, transmission capability, *etc.*,
  ― Aimed at the sensor identity, e.g., cloning, replacing, sybil attack, node fabrication,
  ― Trying to breach the security-related data,
  ― Aiming at the sensor physical integrity, like tampering, chemical, thermal, electromagnetic and similar attacks,
  ― Related to different cases of unforeseen natural catastrophes, emergencies and failures,
  ― Against sensor network and transmission ability (TOE operational environment), like routing misuse, malware, uncontrolled network area accessible to potential intruders, *etc.*,
  ― Attacks causing safety problems—safety-critical faults in the safety-critical equipment,
- Organizational Security Policies (OSPs):
  ― Information flow rules,
  ― Access to asset rules,
  ― Intrinsic safety,
  ― Compliance with standards,
- Assumptions related to the TOE operational environment, like trustiness of administration and intended use of an IT product;
- Security objectives for the TOE and for the TOE operational environment, representing different kinds of security measures, which counter threats, enforce OSPs or uphold assumptions.

All identified elementary issues were expressed by the enhanced generics, which play a role of specification items (in a form of a library) for sensor development, considered one of the CC application domains. The developers choose the right, project-relevant subset of the enhanced generics, refine them by substituting values with parameters values and by adding additional explanations related to the project context. 

The paper presents one of possible examples of the sensors-related generics, selected for a concrete IT product (MethSens). The example is based on the existing design presented in the paper [11]. Please note that only project-relevant items were used.

Examples of enhanced generics can be found in [10,11,12], and their ontological representations in the [12,25]. Enhanced generics defined for the intelligent sensors domain [11] will be implemented in the CCMODE Tools adaptation.

### 2.4. Adaptation of CCMODE Tools to Sensors and Sensors Systems Domain of Applications

The Common Criteria standard and CCMODE Tools can be used for a broad range of IT products. With respect to evidences, no adaptation is required—the configuration according to the EAL requirements and the used external tools are enough. It was assumed that for the validation purpose the Redmine and Testlink tools will not be connected. 

The adaptation is focused on the elaboration of the set of enhanced generics dedicated to intelligent sensors. Most of them were defined in the paper [11,12] (the author encourages reading these papers and taking into consideration the proposed taxonomy and nomenclature of the enhanced generics) and these will be the basis for the CCMODE Tools implementation. In CCMODE Tools generics are included in the knowledge base and the EA-plugin is used to operate on them. EA-plugin is based on ITSDO [12], but uses the Simple Knowledge Organization System (SKOS) [45] technology allowing decision support during the IT security development process, e.g., to propose the most adequate countermeasures to cover the given threat. It will be exemplified during the validation experiment. 

### 2.5. Range of the Validation Experiment

The validation concerns the MethSens sensor which refers to the Methane Early Detection Intelligent Sensor (MEDIS) presented in [11]. The validation is focused on the key issues related to the IT security development of the MethSens:

- CCMODE Tools configuration for the MethSens project;
- IT security modeling of the MethSens with the use of EA-plugin;
- Injecting this model to the MethSens Security Target using GenDoc application.

### 2.6. CCMODE Tools Configuration for the MethSens Project

To start security analyses and the elaboration of evidences, CCMODE Tools should be configured (setup) according to the project needs. The MethSens project setup encompasses the following actions:

- Introducing basic information about the project, like: project type, TOE name and acronym, involved organizations, project roles and actors, subcontractors, *etc.*;
- Configuring connections and working parameters of external modules, like SVN, EA, MS Word, Redmine, Testlink; some tools are optional;
- Selecting the right evaluation assurance level (EAL) for the TOE; components of the EAL are automatically joined to the project; it is possible to perform substitution (replacing one or more components of the given EAL by more rigorous ones) and augmentation (adding one or more component to these implied by the EAL); these two cases are distinguished by “+”, e.g.,: EAL2+; developers can define their own components;
- Defining the IT product life cycle and its phases, processes, actors; some life cycles are predefined in the tool; for MethSens a standard life cycle (with phases: Development, Manufacturing, Operation and maintenance, End of life) was applied;
- Specifying software tools and hardware tools, especially those whose configuration parameters influence the IT product; concerns the “ALC_TAT—Tools and techniques” family requirements [6];
- Specifying tools related to the TOE configuration management; concerns the ALC_CMC, ALC_CMS families [6];
- Setting repository paths pointing at different project artefacts, e.g.,: source files, configuration lists and their items, regulations, *etc.*

**Figure 2:**
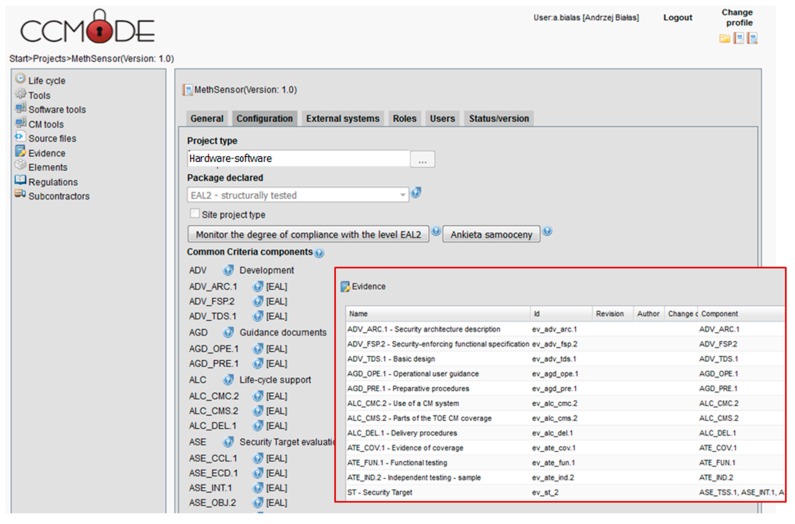
Configuring the MethSens project—EAL2 and its evidences.

Figure 2 shows screenshots examples of the MethSens project configuration using the tool. Please note the horizontal menu on the left and the vertical ones—both presenting different project operations. For the MethSens TOE, EAL2 is applied. The listed EAL2 SARs can be augmented and/or substituted. On the second, red-framed window, evidences for MethSens/EAL2 are listed along with CC components corresponding to them. During the project these evidences will be worked out, *i.e.*, filled in with the contents elaborated by the user or by supporting tools, like EA, EMT. The paper is focused on the security target elaboration (the last position on the evidences list). 

The security target elaboration in GenDoc is preceded by the MethSens security model building, verification and justification with the use of the EA-plugin.

### 2.7. Security Model of the MethSens Device

The security model, after development, justification and verification will be injected into the security target document. The model includes the basic artefacts created during the IT security development: security problem definition (SPD), security objectives (SO), security functional requirements (SFR) and TOE security functions (TSF).

The paper shows how the MethSens security model development is supported by the EA-plugin. Enterprise Architect is a well-known UML modelling tool. The CCMODE EA-plugin extends its functionality:

- To perform CC-related security analyses and modelling for ST (ASE_SPD, ASE_OBJ, ASE_REQ, ASE_TSS families);
- To support the development of evidences dealing with the TOE decomposition (ADV_TDS), interfaces (ADV_FSP), and tests (ATE_FUN, ATE_DPT, ATE_COV) [6].

#### 2.7.1. Security Problem Definition (SPD)—Threats, Organizational Security Policies and Assumptions

The security problem definition encompasses identification of threats impacting the TOE, organizational security policies (OSPs) which the TOE should comply with to be secure, and assumptions for the TOE operational environment. It is preceded by the identification of assets protected by the TOE and different subjects operating on the TOE and its operational environment. To build the security model, the enhanced generics and the relationships between them (like: threatens, counters, covers, upheld, *etc.*) will be used, defined in according to Common Criteria [11,12].

**Figure 3:**
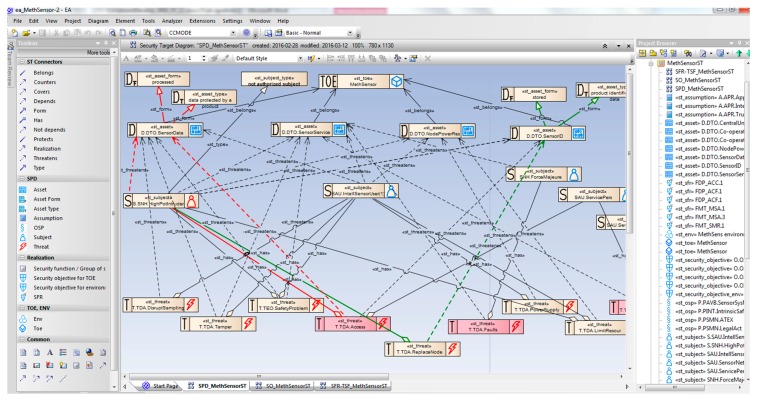
General view of the MethSens security model presented in the CCMODE EA-plugin—Security problem definition diagram.

The general view of the elaborated security model is shown in the middle of Figure 3, more precisely, only a part of the security problem definition is presented (*SPD_MethSensorST* diagram). The parts of the security target, like security objectives, security requirements and TOE security functions are placed on other, logically connected diagrams (*SO_MethSensorST*, *SFR-TSF_MethSensorST*). On the left part of the window, the developer’s toolbox is shown. It includes the UML class stereotypes (rectangles) and relationships (arrows) predefined for the Common Criteria development. They can be drag-and-dropped on the diagram and then refined. On the right part of the figure, the MethSens model artefacts are listed, like diagrams and their elements.

The presented model is rather complicated (seven protected assets marked “D”, six subjects marked “S”, nine threats “T”, four OSPs, and three assumptions “A”, let alone derived security objectives, requirements and functions). It is impossible to present the entire project here. The paper exemplifies the key issue of the security model development only. Please note that to comply with the version 3.1 of Common Criteria, only the generics related to the operation phase of the sensor life cycle are considered in the MethSens project (selected from the MEDIS sensor project [11]). The generics related to the development phase and compliant with the CC ver. 2.x are omitted.

Please note the *TOE:MethSensor* class on the top and four protected assets belonging to it: *DTO.SensorData*, *DTO.SensorService*, *DTO.NodePowerRes*, *DTO.SensorID* (please refer to their descriptions in the paper [11]). The assets: *DTO.CentralUnit*, *DTO.Co-operatEquip(2)*, *DTO.Co-operatEquip(2)* belong to the *ENV:MethSens* class representing the TOE environment (not shown). Each asset class has two optional and auxiliary classes D_F_ and D_T_ representing the assets form (shared, presented, stored, processed, produced, transmitted) and type (e.g.,: data protected by the product, user’s private data, hardware, software, user’s identification data, events log of the product, cryptographic key, and many others). These additional attributes, together with the threats, OSPs, and assumptions properties, are used to propose adequate security objectives by the knowledge engine. 

Two threats related to the TOE will be discussed here on a more detailed level. They represent elementary security problems, whose resolution will be shown later.

##### Example 1

**Figure 4:**
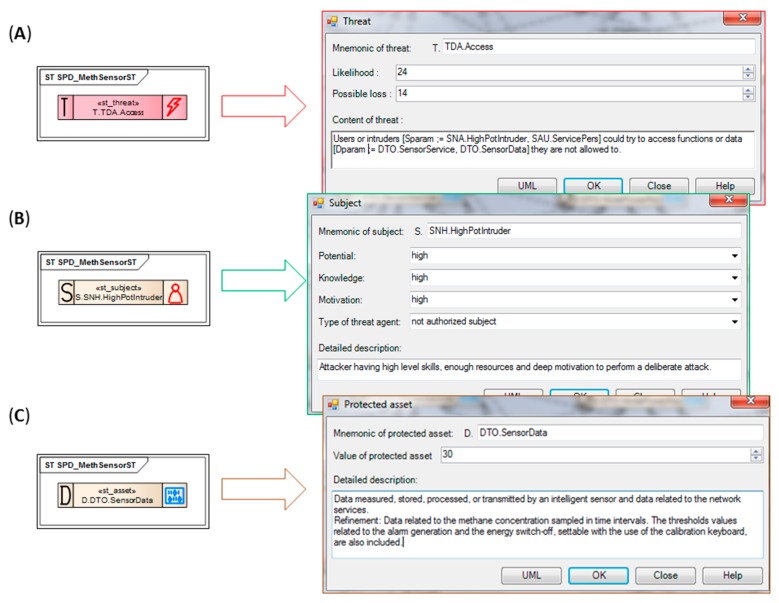
Generics expressing an illegal access attack. (**A**) Threat *TDA.Access* generic and its property; (**B**) Properties of the subject *SNH.HighPotIntruder*; (**C**) Data asset *DTO.SensorData* and its property.

The TOE asset *DTO.SensorData* categorized as D_F_ “processed” and D_T_ “data protected by product” is threatened by the unauthorized *SNH.HighPotIntruder* according to the TOE direct attack *TDA.Access* threat scenario. Figure 3 presents relations between UML stereotyped classes representing generics (marked with thick red lines), like *st_form*, *st_threatens* and *st_has*. Figure 4, in turn, shows properties of these generics. Please note the threat scenario and two risk-related parameters: *Likelihood* of the threat and *Possible loss* when the threat occurs (Figure 4A). Together with *Value of protected asset* (Figure 4C), they allow to calculate risk, *i.e.*, and in the consequence they allow to rank the threats by risk. Using this rank security objectives is more adequate to solve the security problem (will be discussed later).

The *DTO.SensorData* generic includes detailed description as well—please compare it with [11]. The unauthorized *SNH.HighPotIntruder* subject (Figure 4B) has a description expressing the role of the intruder (a threat agent in the CC nomenclature) in the risk scenario, and three parameters *Potential*, *Knowledge*, *Motivation* characterizing the agent. These issues are important when the TOE vulnerability is assessed by evaluators (AVA_VAN family [6]). 

##### Example 2

**Figure 5:**
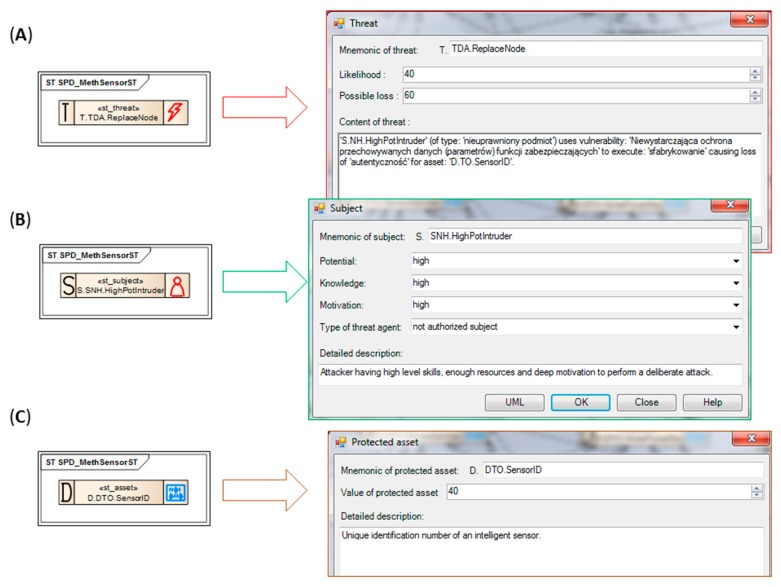
Generics expressing an attack against the sensor identifier. (**A**) Threat *TDA.ReplaceNode* generic and its property; (**B**) Properties of the subject *SNH.HighPotIntruder*; (**C**) Data asset *DTO.SensorID* and its property.

The second example is very similar and concerns an attack against the sensor identifier, which leads to spoofing. *DTO.SensorID* categorized as D_F_ “stored” and D_T_ “product identification data” is also threatened by *SNH.HighPotIntruder* according to the *TDA.ReplaceNode* threat scenario. Please note the relations in Figure 3 (marked with thick green lines) between classes representing generics. Figure 5 shows properties of these generics.

The above mentioned threat generics were selected from the list of items predefined for sensors. The EA-plugin allows to create threat generics semi-automatically, based on dialogue forms. The developer is asked about different features of the threat, threatened asset and threat agent. On this basis a complete scenario is made and, finally, the generic is placed on a diagram. This option will not be discussed in this paper. 

#### 2.7.2. Security Objectives (SO)—Solving the Security Problem

Security objectives express the proposed security measures by means of semiformal descriptors—enhanced generics. To build the SO specification, one can use ready-made generics from the library or generics defined semi-automatically on the basis of threats, OSPs, assumptions (only for the operational environment), subjects and assess their properties. This selection is based on the CCMODE ontology (SKOS) implemented within EA-plugin.

**Figure 6:**
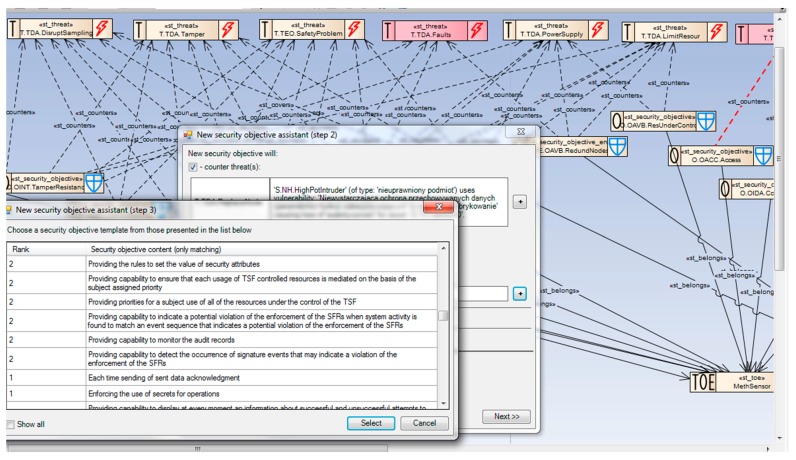
Security objectives selection based on the ontology-produced rank list.

**Figure 7:**
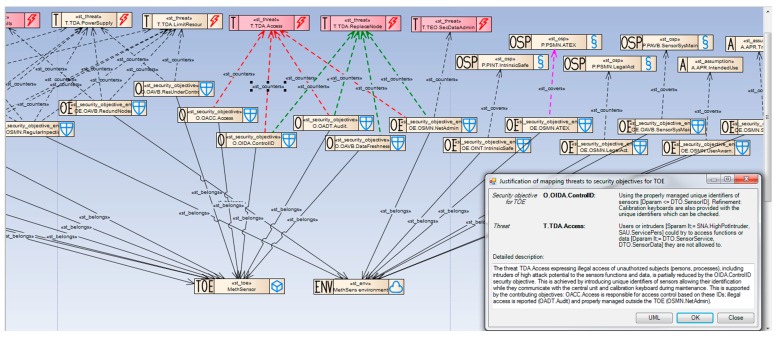
General view of the MethSens security model presented in the CCMODE EA-plugin—a part of the security objectives diagram.

Each security objective generic has associated different properties related to its character (means/ways to solve a security problem, e.g., access control) and properties inherited from the countered threat, enforced OSP, upheld assumption, related assets (specified by D_T_, D_F_) and subjects (subject_type). Using the set of properties, the SKOS-type ontology proposes a ranked list of security objectives to be used to solve the given problem. Figure 6 shows the security objective selection supported by the SKOS ontology implemented in EA-plugin. Please note some propositions, some more preferable (score = 2), some less (score = 1). Figure 7 shows the *SO_MethSensorST* diagram presenting how the security problem is solved by selecting right security objectives for the TOE and its environment. Generally, the TOE objectives (marked “O”) counter threats and/or cover OSPs. The TOE environment objectives (marked “OE”), which support the TOE objectives, counter threats and/or cover OSPs and/or uphold assumptions [6]. 

##### Example 3

This example is a continuation of Example 1. The *TDA.Access* threat is countered by three TOE objectives:

- *OACC.Access. The sensor must control access of connected entities*;
- *OIDA.ControlID. Using the properly managed unique identifiers of sensors [Dparam<=DTO.SensorID]. Refinement: Calibration keyboards are provided with unique identifiers which can be checked*;
- *OADT.Audit. The sensor must audit attempts to undermine its security and should trace them to the associated entities*;

and supported by the TOE environment objective:

- OSMN.NetAdmin. Network administration and security policy procedures implementation.

Note: Sometimes the refinement is emphasized by the underlined word “*Refinement:*”.

Particular *st_counters* relations are marked by red dashed arrows. Each of the countering relations should be justified. The justification is shown in the right bottom part of the window (Figure 7).

##### Example 4

The *TDA.ReplaceNode* threat (discussed in the Example 2) is countered in a similar way by three TOE objectives: *OIDA.ControlID*, *OADT.Audit*, *OAVB.DataFreshness*, supported by the TOE environment objective *OSMN.NetAdmin*. Particular *st_counters* relations are marked by green dashed arrows.

##### Example 5

The *PSMN.ATEX* policy (OSP) is covered by the TOE environment objective *OSMN.ATEX*. The relation *st_cover* (requiring justification as well) is marked by the purple dashed arrow. Please note other OSPs and assumptions (marked “A”) covered by the TOE environment objectives.

#### 2.7.3. Security Functional Requirements (SFR)—Semiformal Representation of the Security Objectives

Security objectives describe the solution of the security problem in an informal developer’s language, with the use of enhanced generics. Generics are semiformal but they contain informal descriptors of security measures, which may be subjective, because descriptors are created by particular developers. To describe any security measure in a non-subjective, unified way, semiformal SFR components are defined in the Common Criteria standard.

Each security objective should be translated (mapped) to one or more SFR components selected from Part 2 of the Common Criteria standard [6].

**Figure 8:**
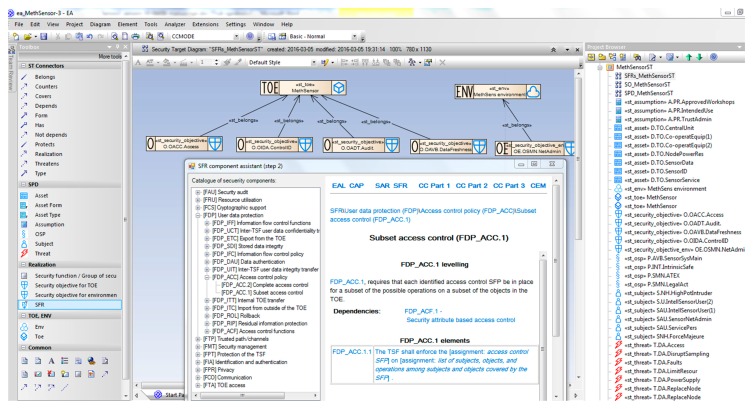
MethSens security model presented in the CCMODE EA-plugin—mapping security functional requirements to security objectives.

Figure 8 presents the mapping process of security functional requirements to security objectives. All SFRs are implemented in the CCMODE knowledge base in the form of a hierarchical tree: classes-families-components, *i.e.*, in the same way as they are placed in the Common Criteria SFRs catalogue ([6]/Part 2). As it was mentioned earlier, the given security objective has many properties associated, including means and ways of the security problem solution, e.g., access control, inherited properties of a countered threat or an enforced OSP, even inherited properties of involved assets and subjects. Based on these security objectives, security functional components are proposed, selected from the hierarchical tree.

To the given security objective a right component is assigned with some dependent components (if they exist and are required in the given circumstances). Please note that for the *OACC.Access* objective the *FDP_ACC.1* (Subset access control) component is selected. It has a dependent component *FDP_ACF.1* (Security attribute based access control) which requires its own dependent component to be selected, *i.e.*, *FMT_MSA.3* (Static attribute initialization) [6]. This selection is strongly supported by CCMODE Tools. The developer selects some dependencies for implementation and rejects others as irrelevant in the given circumstances.

##### Example 6

**Figure 9:**
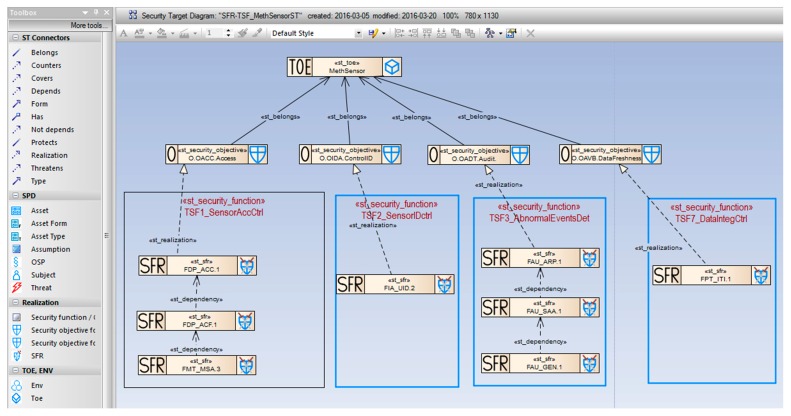
General view of the MethSens security model presented in the CCMODE EA-plugin—security functional requirements grouped by TOE security functions.

Figure 9 shows the *SFR-TSF_MethSensorST* diagram presenting several security objectives expressed by SFR components:

- *OACC.Access* expressed by: *FDP_ACC.1 (FDP_ACF.1, FMT_MSA.3)*; explained above;
- *OIDA.ControlID* expressed by: *FIA_UID.2*, requiring user identification before any action; it has no dependencies;
- *OADT.Audit:* expressed by: *FAU_ARP.1* (Security alarm when security violation occurs); it has a dependent component *FAU_SAA.1* (Potential violation analysis) which, in turn, has another dependent component *FAU_GEN.1* (Audit data generation);
- *OAVB.DataFreshness* expressed by: *FPT_ITI.1* (Inter-TSF detection of modification) which has no dependencies.

When all security objectives have SFRs mapped and their dependencies are solved, the security functional requirements specification is ready. At this stage the security model needed for the protection profile is finalized. For the security target the next step is required. The SFRs are grouped and assigned to the defined TOE security functions (TSFs) to be implemented in the IT product at the claimed EAL level. This way the TOE summary specification (TSS) is worked out. It is shown on Figure 9 as well. The particular TSFs are expressed by rectangles embracing their relevant SFRs. 

##### Example 7

Figure 9 shows the *SFR-TSF_MethSensorST* diagram presenting the TOE security functions meeting a particular SFRs group:

- *TSF1_SensorAccCtrl* meeting the requirements included in the SFRs: *FDP_ACC.1, FDP_ACF.1* and *FMT_MSA.3*;
- *TSF2_SensorIDctr* meeting the requirements included in the SFR: *FIA_UID.2*;
- *TSF3_AbnormalEventsDet* meeting the requirements included in the SFRs: *FAU_ARP.1, FAU_SAA.1 and FAU_GEN.1*;
- *TSF7_.DataIntegCtrl* meeting the requirements included in the SFR: *FPT_ITI.1*.

The TSS preparation completes the security model elaboration needed for the security target.

### 2.8. MethSens Security Target Elaboration

**Figure 10:**
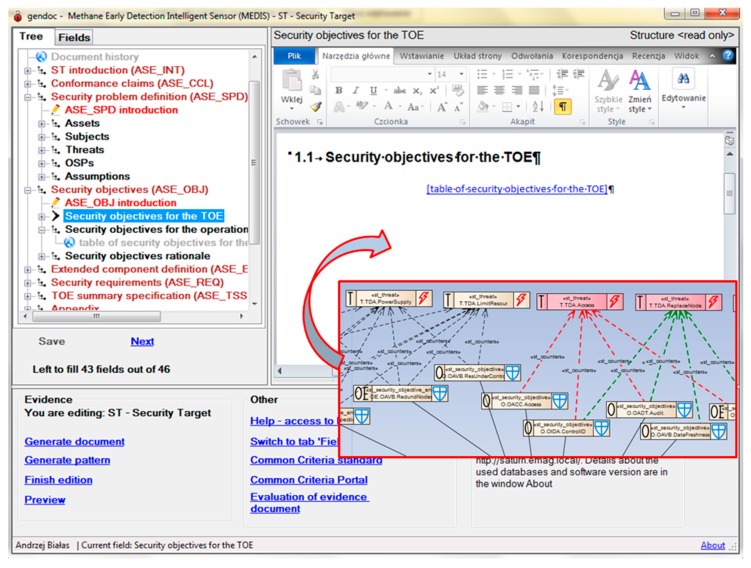
Security model transferred to the elaborated evidence presented in the CCMODE GenDoc application.

The security model of the sensor can be injected into the right sections of the security target evidence elaborated on the pattern basis. Figure 10 shows a part of the model representing security objectives to be injected to the relevant section of the ST. Please note the structure of the pattern (and the evidence) on the left.

**Figure 11:**
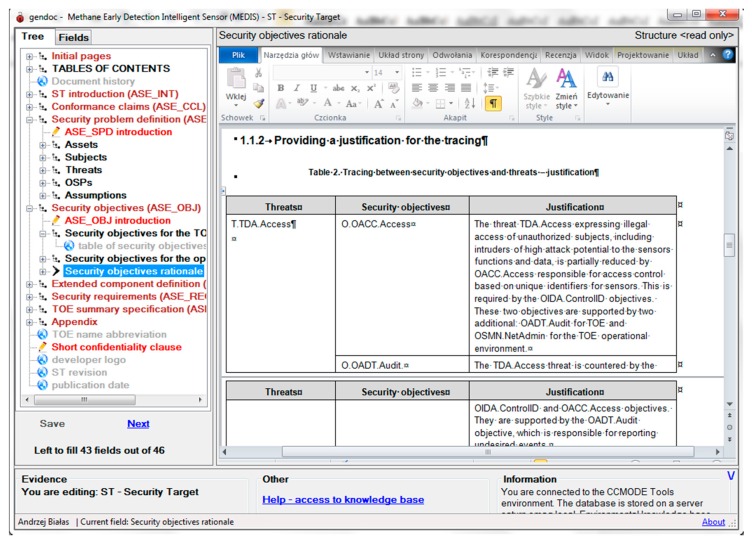
Part of the security target automatically elaborated on the basis of the security model with the use of the CCMODE GenDoc application.

Figure 11 presents the security objectives rationale section of the ST. Please note that justifications provided during the security model elaboration (see Figure 7) were automatically placed in the ST document. The ST elaboration is an iterative process with the use of the EMT, EA and GenDoc applications.

During the evidence elaboration the developer can check how this evidence will be evaluated in the security testing lab. This checking is called here self-evaluation, CCMODE Tools includes the full implementation of the Common Evaluation Methodology for Information Technology Security (CEM) [46].

According to the Common Criteria standard each SAR component includes three kinds of elements:

- Developers’ action elements (marked “D”) expressing what the developers should provide as evidence;
- Contents and presentation elements (marked “C”) showing what kind of requirements the evidence should meet;
- Evaluators’ action elements (marked “E”) showing how the evaluator should verify the provided evidence.

**Figure 12:**
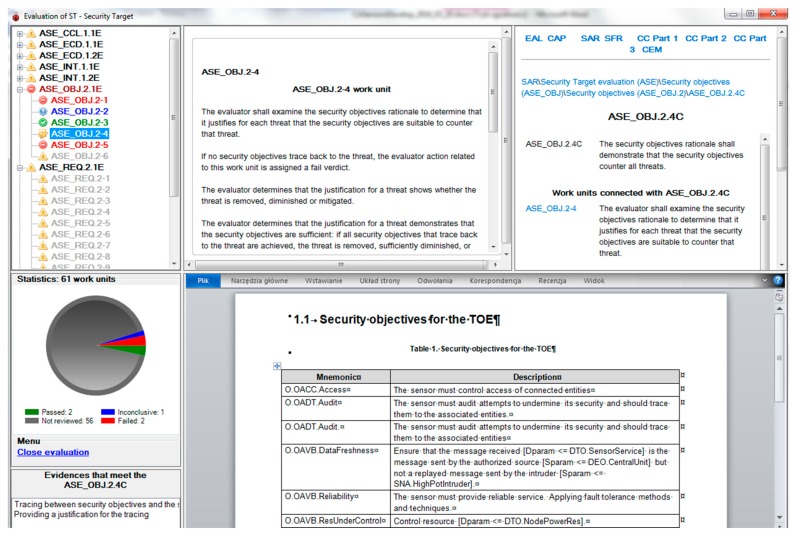
Self-evaluation of the MethSens security target presented by the CCMODE GenDoc application.

The evaluation application is presented in Figure 12. On the left side there is a tree with the assessed work units implied by particular evaluators’ actions elements. Each of 61 work units should have a verdict assigned with justification. Possible verdicts are: pass, fail, inconclusive. They are shown on the statistics diagram. The assessed section of the security target is displayed in the central bottom part. Above, the corresponding work unit (on the left) and the relevant SAR component (on the right) are shown—precisely, the contents and presentation element. Please note that the evaluator is equipped with complete information allowing to assign a verdict.

The statistics show progress of the evaluation process. This application is designed for evaluation labs, although developers can use it optionally.

### 2.9. Other Evaluation Evidences for the MethSens Device

Other required evidences implied by the EAL are elaborated in the same way but this issue is beyond the scope of the paper. These evidences cover the TOE development process and concern:

- TOE architecture, its functional specification, design, security policy, implementation (ADV class);
- Life cycle definition, configuration management, product delivery, development process security, used tools and their options (ALC class);
- Tests specification, test depth and coverage (ATE class);
- Product manuals and procedures (AGD class);

Vulnerability assessment of the TOE and its development site (AVA class).

## 3. Results and Discussion

The Common Criteria standard presents a renowned and matured security assurance methodology used for different categories of IT products and systems. The most popular CC certified products are: smart cards and devices related to them, network devices, multifunction devices, operating systems, boundary devices, and digital signature devices [7]. The developers representing these CC application domains are very experienced, and equipped with supporting methods and tools. There are some specific IT products and systems which are problematic for their developers, and the number of evaluated/certified solutions is very low. This is due to existing barriers and generally low experience in the use of Common Criteria by IT product developers. Simply, the developers find it difficult to transform the specific technical language of the IT product domain to the specific language of Common Criteria which is used to elaborate the evaluation evidences as the input in the certification process. The intelligent sensors and sensors systems domain is an example of such IT products domain (a niche domain) and the use of the CC methodology in it is very scarce (Section 1.2). 

Sensor developers need some help, including the knowledge how to understand the CC nomenclature and to use it as well as supporting tools and design patterns to overcome most of the existing difficulties. The CCMODE Tools suite [15] offers such support for typical IT products or systems. This is a specialized, Common Criteria compliant, patterns- and knowledge engineering-based, computer-aided engineering system. There is no straightforward answer to the question whether this suite can be helpful in the sensor domain. To settle it, the validation experiment was planned and performed. The aim of the validation experiment is to assess:

- If it is possible to adapt this suite for the specific sensors application domain in order to support sensors developers;
- Whether the extended customized suite could be useful for intelligent sensors and systems developers.

### 3.1. Validation Context

This assessment is performed through the validation experiment, discussed in Section 2. The main experiment was preceded by a short presentation of the customized tool and the context of the experiment. The CCMODE Tools suite was configured to enable the following:

- The management of a sensors-related project in the assumed life cycle (development, manufacturing, operation & maintenance, end of life) but the validation was focused on the development phase;
- The EAL2 for the sensors project;
- Security analyses and modelling (EA-plugin);
- Semi-automatic generation (GenDoc/MS Word) of evaluation evidences; the validation was focused on the basic evidence, *i.e.*, security target;
- Self-assessment of the evidences;
- The preparation of a knowledge base;
- The establishment of a repository of project artefacts (SVN).

The test manager (Testlink) and the bug tracking tool (Redmine) were not used in the experiment, but generally there is no obstacle to use it in sensors projects. The validation experiment was based on the MethSens sensor with well identified project data, based on the MEDIS sensor project described in [11]. 

EAL2 was claimed for the *MethSens* device, as mentioned above. The validation experiment is focused on the security target (ST) elaboration, as the most important evaluation evidence and the basis for other TOE-related evidences. The security target is the same (the same ASE class components) for any of the EAL2-EAL7 levels. From the perspective of the validation experiment, the claimed EAL is irrelevant, because the EAL does not influence the TSFs shape, but the developed TSFs should be implemented on the claimed EAL. The claimed EAL influences the TOE-related evidences—not discussed, only mentioned in the paper. Generally, the EAL selection for the IT product is a much more complicated issue. Different factors may influence this selection:

- Law enforcement—some products, especially for government-like applications, should have the given EAL (an arbitrary decision);
- Market requirements (trade-off based on market-related factors).

When EAL is selected for an IT product which is to be placed on the market, the trade-off between cost and assurance should be done. A higher EAL means higher rigour is applied (more assurance components which include more rigour), and the higher rigour means a higher cost of development and evaluation. In addition, the product range and complexity influence this cost. Customers may not accept such a high price of the product. 

On the other hand, the customers may refuse to acquire IT products for responsible applications (high value of protected assets, high risk) with too low claimed assurance levels. The customers may not accept such products because they do not meet their needs.

#### Conclusion 1

The range of the experiment encompassed all typical developers’ activities. The assumed EAL2 is rather low, though according to the author’s subjective opinion, it is adequate (security assurance *vs.* costs) for many sensors applications. Apart from the security target (ASE), it implies 10 evidence documents related to: the sensor architecture (ADV_ARC), decomposition (ADV_TDS), interfaces (ADV_FSP), configuration management (ALC_CMC, ALC_CMS), delivery procedures (ALC_DEL), preparative procedures (AGD_PRE), operational user guidance (AGD_OPE), functional tests (ATE_FUN), test coverage (ATE_COV), which ought to be delivered by the developers. From the research perspective the most difficult and interesting is the security target, others concern rather routine and practical issues. 

The object of the validation experiment, *i.e.*, MethSens, is representative but not trivial. MethSens comprises many diversified security issues: diversified architecture, assets, threats, policies, objectives, requirements and functions. For this reason the validation can be considered comprehensive and representative for the discussed domain of application. 

### 3.2. Range of Development Suite Customization

Considering the evidences patterns, there is no need to perform any adaptation, because patterns are implied by assurance components and do not depend on the category of the IT product or system. The CCMODE offers transparent, domain-independent patterns.

A more complex situation emerges with specification means patterns. There are two groups of them. The first group encompasses security functional requirements which are defined in the CC standard and are directly implemented in the CCMODE knowledge base. No adaptation is needed either.

The second, very diversified group of specification means, depends strongly on the IT product category. There were no sensors-specific generics implemented in the CCMODE. There was a need to define and include in the knowledge base the generics specific for intelligent sensors and sensors systems. Such generics were identified in the author’s previous work [11]. During the validation these generics were verified, sometimes modified (all changes were marked in violet in Figure 3, Figure 7) and introduced into the CCMODE knowledge base. This way CCMODE Tools was prepared to be used in a new domain of application. 

#### Conclusion 2

The adaptation of CCMODE Tools was restricted to the implementation of enhanced generics specific for sensors. The specialized domain library of generics was implemented.

### 3.3. Course of Validation Experiment

The paper does not present the elaboration of all EAL2 evidences but only the most important and difficult elements of the security target. The developers usually have trouble with security analyses, modelling, rationale, and with the right contents and coherency of evidences. The first issue is solved by applying the EA-plugin, the second by evidence patterns and GenDoc, supported by other CCMODE Tools components.

All IT security development process stages were exemplified by means of the EA-plugin. In addition, it was shown how to specify particular issues using enhanced generics or CC components, how to solve the security problem, how to express the problem solution by security functional requirements, how to work out security functions, and how to justify particular development stages. 

The developed security model was injected into the security target by the GenDoc application. Each change within the model was also illustrated on line in the elaborated evidence. Each evidence is implied by its pattern and the pattern is compliant with the corresponding security assurance requirements. This way the developer, guided by software, is fully focused on the IT product and does not preoccupy with the composing evidences the scratch. It was shown on the example related to the security objectives justification.

#### Conclusion 3

The validation experiment encompasses all activities considered difficult or laborious by developers. These activities were supported by the software tool which made these activities much more easier. The data common for all evidences, e.g., different names, identifiers, are managed by EMT. Project artefacts are versioned and stored by SVN. The analyses/modelling/justifications are supported by the EA-plugin. The evidences are semi-automatically generated by GenDoc.

### 3.4. Self-Assessment—Useful Optional Step

The validation experiment pays attention to an optional activity—the developer is able to perform the evaluation of his/her work in the similar way as it will be done later in the evaluation lab. It is called here self-assessment and it usually encompasses issues which are difficult and require proper interpretation. The developer has direct access to the relevant evaluation work units and can see how the given issue will be assessed by evaluators according to CEM [46]. It was shown on some examples related to the security objectives rationale.

#### Conclusion 4

The validation experiment encompasses an optional step related to the self-assessment of the given issue of the elaborated evidence. This is a general, non-sensor specific functionality of CCMODE Tools. The validation experiment stimulates the supplementation of CCMODE Tools with elements allowing its application in the sensor domain. The IT security development and the TOE development processes are fully supported by this suite. 

## 4. Conclusions

The paper concerns security assurance in the context of intelligent sensors and sensor systems. Security assurance is understood here according to the paradigm expressed by the Common Criteria methodology. This methodology is mature, broadly used, but still improving, because some parties consider it difficult, laborious, costly and generating huge documentation. The discussion seems to be endless, but in fact, no other methodology is able to replace Common Criteria now, or even to simplify it. Efforts to enhance it, to support it, to make it more friendly for developers seem to be still purposeful. This paper follows this statement.

There are about 2089 CC-certified IT products and 173 registered protection profiles now [7] but their distribution by product categories is irregular. Some application domains can be considered niche domains. Sensors are one of them. The answer to the question “why?” may be complicated. The causes may be: low market demand, no strong requirements implied by laws, difficulties in applying the CC methodology in this specific domain of application, low security awareness, low CC-related knowledge among the sensors developers, *etc.*

Additionally, the review of the research field (Section 1.2) shows that the Common Criteria applications for sensors and sensors networks are scarce but they embrace very important cases, like: healthcare systems, aircraft health monitoring systems, safety-critical assets distribution systems, transport, including motion sensors of digital tachographs, and SCADA-related products.

This shows that the sensors systems development is an emerging CC domain of application. There exist different barriers in such emerging domains. To overcome them, it is necessary to disseminate knowledge about Common Criteria in the sensor developer community, provide the developers with dedicated methods, design patterns and tools. 

At the beginning of the research the author assumed that it is possible to design a Common Criteria compliant security assurance suite dedicated to the development of intelligent sensors and sensors systems. The author’s motivation was to help the sensors systems developers in the implementation of the Common Criteria methodology.

The objectives of the research presented in the paper were:

- To adapt the general-purpose CCMODE methodology and its supporting tools to the needs of the intelligent sensors and sensors networks domain;
- To disseminate CC-related knowledge in this domain.

### 4.1. Research Findings and Implications

To assess the feasibility of the above mentioned concept the validation experiment was performed. It was assumed that:

- The development suite dedicated for sensors will be based on the general purpose, ready-made platform—CCMODE Tools;
- The feasibility study will be based on the representative sensor MethSens/EAL2.

The validation experiment embraces three basic Common Criteria processes, though only some essential issues were exemplified in the paper. 

For the IT security development process, the most important and difficult activities were performed to assess the concept feasibility:

- The security modelling and rationale—supported by the EA-plugin;
- The patterns- and tools-based semi-automatic generation of evidences (replacing the old laborious, manual elaboration of evidences)—the MethSens security target generated by the GenDoc application;
- The sensors-specific security specification work out—a set of enhanced generics was predefined in the CCMODE knowledge base and some of them were used to specify the MethSens security model.

The TOE development process embraces the elaboration of different evidences documents. They were generated as empty documents on the basis of patterns of the EMT application according to EAL2 (Figure 2). The TOE development process evidences were not fully exemplified because they are less complicated than the above discussed security target.

The TOE evaluation process is performed in accredited evaluation labs. CCMODE Tools includes the CEM implementation which is able to perform similar evaluation, but understood as self-evaluation. The developers can check optionally how certain evidences or their parts will be evaluated with the use of CEM work units.

The conclusion is that CCMODE Tools can be adapted to the sensor domain of application mainly by the extensions of the specification means library (sensor-specific enhanced generics). The adapted suite can support sensor developers in all development activities. The knowledge acquired during the validation experiments can be used by sensor developers in their projects. 

### 4.2. Paper Contribution and Future Research

The paper contribution embraces the adapted patterns-based computer-aided methodology for the sensors development. This methodology has an innovative character and goes beyond the sensors domain. It concerns any IT product development, improves the product quality and security assurance. It is achieved by:

- Better project management—thanks to the Common Criteria compliant, IT security development framework equipped with the domain knowledge base, design patterns, defined development processes, life cycle models, supporting tools, and communication facilities;
- Automation of IT product development process—complex and laborious activities are supported by software tools; this way it is possible to achieve the project reusability and other advantages similar to those gained in the CAE systems.

The CCMODE Tools suite was used for several kinds of IT products, like: data diodes [47], different sensors, RFID devices. The CCMODE Tools suite is very useful in the development processes, though it needs more validation in different application domains. The CCMODE Tools methodology is still enhanced based on the feedback from running projects and validations. 

The author is involved in research going beyond the Common Criteria methodology, especially research on new security assurance paradigms, including the Trustless Computing initiative [48]. It is “a global initiative for the creation, from existing open components, of the World’s most user trustworthy general-purpose end-2-end computing service platform, lifecycle, open ecosystem and international certification body, aggregating world class partners and advisors.” The author’s research objective is to consider how to extend the CCMODE methodology by new security assurance paradigms. 

## Figures and Tables

**Table 1:** Security target/protection profile patterns.

Pattern Acronym	Pattern Name	Description
STp	Security Target pattern	Structure and contents of the security target (ST).
laSTp	low assurance Security Target pattern	Structure and contents of the low assurance security target used for EAL1
PPp	Protection Profile pattern	Structure and contents of the protection profile (PP)
laPPp	low assurance Protection Profile pattern	Structure and contents of the low assurance protection profile used for EAL1
SSTp	Site Security Target pattern	Structure and contents of the site security target (SST).

**Table 2:** Evaluation evidence patterns related to the development environment (site).

Pattern Family	Name	Description
ALC_LCDp	Life-cycle model definition patterns	Presents high-level description of the TOE life-cycle and provides a framework for the entire development environment.
ALC_DVSp	Development security patterns	Specifies physical, procedural, personnel, and other security measures to be used in the development environment to protect the TOE and its parts.
ALC_CMCp	Configuration management (CM) capabilities patterns	Describes in detail the management of configuration items and enforces discipline and control in the processes of refinement and modification of the TOE and the related information.
ALC_CMSp	Configuration management scope pattern	Shows how to specify items to be included as configuration items and hence controlled by the above CM capabilities.
ALC_TATp	Tools and techniques patterns	Is responsible for control tools, their options and techniques used in the development environment (programming languages, documentation, implementation standards, runtime libraries, different equipment, *etc.*).
ALC_DELp	Delivery patterns	Describes the secure transfer of the finished TOE from the development environment into the responsibility of the user.
ALC_FLRp	Flaw remediation patterns	Concerns the detected security flaws that should be traced and corrected by the developer.

**Table 3:** Evaluation evidence patterns related to the IT product.

Pattern Family	Name	Description
ADV_ARCp	Security Architecture patterns	Describes the security architecture of the TOE security functions to show if they achieve desired properties (how to use architectural properties to better protect security functions).
ADV_FSPp	Functional specification patterns	Describes the TOE security functions (TSFs) interfaces (TSFIs) which contain the means for users to invoke a service from the TSF (by supplying data that are processed by the TSF) and the corresponding responses to those services invocations.
ADV_TDSp	TOE design patterns	Provides context for the TSFs description and describes the TSFs. The TOE decomposition is specified on different levels of detail (subsystems, modules) with respect to the applied rigour (EAL).
ADV_IMPp	Implementation representation patterns	Expresses how the TSFs are implemented (software/firmware/hardware design language source code, hardware/IC diagrams, layouts).
ADV_INTp	TSF internals patterns	Addresses the assessment of the TSFs internal structure. Well-structured TSFs are easier to implement and have fewer flaws and vulnerabilities.
ADV_SPMp	Security policy modelling patterns	Provides additional assurance from the development of a formal security policy model of the TSF and helps to gain correspondence between the functional specification and this security policy model.
AGD_PREp	Preparative procedures patterns	Presents how the TOE has been received and installed in a secure manner as intended by the developer.
AGD_OPEp	Operational user guidance patterns	Shows how to prepare written material intended for all types of users of the TOE in its evaluated configuration.
ATE_FUNp	Functional tests patterns	Enforces the right specification, execution and documentation of tests.
ATE_COVp	Test Coverage patterns	Helps to demonstrate that the above mentioned TSFIs are properly covered by tests.
ATE_DPTp	Test Depth patterns	Helps to demonstrate that the specified TOE design elements (subsystems, modules) are properly covered by tests.
ATE_INDp	Independent testing patterns	The ATE_IND evidences are elaborated by evaluators. This evidence is used to perform the tests provided by the developer and to perform additional tests defined by evaluator.
